# Lecture 1—Justification of the Value of Clinical Pharmacy Services and Clinical Indicators Measurements—Introductory Remarks from a Traveler on a 40-Year Wayfaring Journey with Clinical Pharmacy and Pharmaceutical Care

**DOI:** 10.3390/pharmacy6030056

**Published:** 2018-06-27

**Authors:** Richard H. Parrish, Lita Chew

**Affiliations:** 1 St. Christopher’s Hospital for Children, American Academic Health System, Philadelphia, PA 19134, USA; 2 School of Pharmacy, Virginia Commonwealth University, Richmond, VA 23298, USA; 3 Department of Pharmacy, National University of Singapore, Singapore 117543, Singapore; Lita_CHEW@moh.gov.sg; 4 Ministry of Health, Pharmacy Services, Government of Singapore, Singapore 117543, Singapore

**Keywords:** clinical pharmacy, pharmaceutical care, commodification, patient-driven

## Abstract

Without question, health care delivery, and clinical pharmacy’s purpose in it, is changing rapidly all over the world. Pharmacy’s place in the new health care environment is ensured only to the extent that the purpose of pharmaceutical care is understood and transmitted to the global structures of these developing organizational patterns and paradigm shifts. While the current trend toward commodification of illness and treatment seems to be driving efforts to consolidate the economic factors of pharmaceutical distribution, a new type of practice—patient-driven health care—has continued to shape the interactions of pharmacists and patients all over the world. A thorough understanding of the above factors involved in pharmacy’s history, present, and future are necessary for clinical practice preparation, as well as for value justification. How clinical pharmacy will succeed in this kind of social and economic milieu is precisely why this series of lectures and roundtables will help us embrace many of the vexing issues that clinical pharmacy administrators and practitioners face in daily practice.

## 1. Introduction

Without question, health care delivery, and clinical pharmacy’s purpose in it, is changing rapidly all over the world. The incidence and prevalence of corporate mergers and acquisitions make the determination of ownership of the factors for producing health care very difficult. Pharmacy practice has been a part of, as well as felt, the impact of vertical and horizontal integration. Hospital pharmacies have become health system focused. Community pharmacies have lost business to mail order divisions of pharmacy benefit managers or large drug store chains. Moreover, pharmaceutical companies have become sensitive to pharmacoeconomics by increasing research budgets to demonstrate the clinical and economic value of their products and services prior to marketing. New types of prescribers—nurse practitioners, physician assistants, optometrists, physical therapists, and, yes, clinical pharmacists—are past the point of pilot studies in many areas of the world. My recent practice on the visceral surgery unit of a large tertiary care hospital in inner city Edmonton, Alberta, is one personal example where clinical pharmacists are practicing on a scope consistent with the general practice of medicine.

## 2. Clinical Pharmacy’s Strengths in the Care Setting

Pharmacy’s place in the new health care environment is ensured only to the extent that the purpose of pharmaceutical care is understood and transmitted to the global structures of these developing organizational patterns and paradigm shifts. A language of leadership about pharmacotherapy is necessary for clinical pharmacists to advocate for the drug-therapy related needs of patients. The public continues to hold pharmacists in high regard in terms of ethical standards and honesty. Knowledge of these influences and strategies for optimizing the contributions of pharmacists in patient care via the new practice methodology requires a thorough understanding of macro level issues like health services delivery, population health, and predictive analytics, financing, marketing, and pharmacoeconomics. What must be brought to bear on these larger institutional players in health care is the fact that no professional systematically follows and takes responsibility for drug therapy. The phenomenon of drug-related morbidity and mortality is estimated to cost the United States over $200 billion annually [1]. To my knowledge, the clinical and economic costs for Singapore have yet to be estimated. It has been estimated that pharmacists practicing patient-centered care can increase the number of patients who receive optimal outcomes by 41%, reduce the cost of drug-related illness by almost 60%, and contribute to almost 120,000 lives saved per year! Over 200,000 pharmacists could be supported out of the surplus created if the profession took responsibility for addressing drug-related morbidity and mortality. A focus on patient outcomes could re-direct attention from financing schema to care giving. Caring means being present in order to listen to and know the patient. Perhaps a secure connection with Skype or another webinar-based functionality could work here.

While the current trend toward commodification of illness and treatment seems to be driving efforts to consolidate the economic factors of pharmaceutical distribution, a new type of practice—patient-driven health care—has continued to shape the interactions of pharmacists and patients all over the world. However, many patients receive their medications by mail. Many patients act as their own pharmacist, trading the cost reductions inherent in mass production for knowledge of how the medication will affect them, what outcomes they should expect, and what they will do if problems arise. Prudent benefit managers understand the gap between merely receiving discount pharmaceuticals and the required knowledge and intelligence about medication-use, and are incorporating into their plans a role for drug therapy problem solvers. These pharmacists are responsible for educating and monitoring the process of care for identified populations of patients at risk of developing drug therapy problems (DTPs). Whether the trend toward so-called “carve-out” care (i.e., disease state management) or more holistic care will dominate remains to be seen. However, both modes will continue to influence care delivery.

What is important for pharmacy (and health care executives) at the present time is to appreciate the economic and political factors driving health care delivery, and capitalize on its current strengths in the market. It is vital for those associated with health care in any way to realize that over 80% of all care worldwide is self-care [2]. In all areas of the world, pharmacy’s strengths lie in three salient observations: (1) the frequency of visits made to community pharmacies; (2) the high level of trust patient have for the pharmacists who care for them; and (3) the evolving scientific research on patient outcomes, both individually and in the aggregate [3,4]. While organized pharmacy in the US may have elaborate political strategies for ensuring pharmacy’s future in the health care delivery process, what pharmacists do with individual patients will make the difference for professional survival.

A thorough understanding of the above factors involved in pharmacy’s present and future are necessary for clinical practice preparation, as well as for value justification. The causes of drug therapy problems are related directly to breaks in the caring process. Stewart and colleagues describe the care process as being comprised of six elements [5]. In order to render care, we must take time to explore both the disease (the professional definition) and the illness (the patient’s definition) so that the patient is understood. This is the basis of the therapeutic relationship. Being understood is important. Secondly, for a complete picture of the patient’s reason for seeking consultation, we must attempt to understand the whole patient, not as a diabetic or hypertensive, but how their life affects and is affected by their condition. We must take into account all of the patient’s perceptions, emotions, background, physical presentation, and relationships in order to know patients “in themselves” [6]. Our natural curiosity about the patient is vital to the formation of trust because our patients feel we are listening and want to help them. The third component is finding common ground. We cannot be everything we want to be, in life, health, or illness. Often, negotiating with the patient about the limitations inherent in their health condition expands our understanding of how the patient will actually live with our recommendations when they leave the office or pharmacy. Moreover, patients present with problems that confront the limitations of our own abilities. Being open to agreements that the patient will commit to involves compromise. Patients who acquiesce to a professional solution will not always live it out if doing that involves breaking habits of living. By finding common ground, decisions about the nature and seriousness of problems, goals of treatment in terms of expected outcomes that matter to the patient, and the roles of each are reached by mutual agreement for mutual gain. The therapeutic process as a true healing modality is based on the teleology inherent in the “goal-directedness” of living action [7].

The fourth component of the caring process incorporates prevention and health promotion. In the context of clinical pharmacy, it is estimated that between 50 and 80% of drug therapy problems are preventable [8,9]. In the aggregate, this proactive approach is worth over $50 billion USD annually. To the individual patient, it could mean survival or death. Patients must understand the consequences of the actions, and take responsibility for success or re-negotiation. Prevention and health maintenance are a major part of clinical pharmacy and pharmaceutical care, without which only reaction to presenting symptoms could occur. Often, clinical pharmacists must comfort and reassure patients, expressing empathy, congruence of purpose, respect, positive regard, and concern. As Stewart et al. pointed out, a trusting relationship, the foundation for all interchanges between patient and practitioner, must be constantly nurtured in order for it to continue. The key to a sustained relationship is self-awareness. As health professionals, clinical pharmacists have a mandate to be close to patients; that closeness fosters a sense of connectedness to others that may have been broken through their suffering. Finally, we must be realistic with our expectations of patients and of ourselves with particular reference to time, accessing resources, team building, and stewardship.

How clinical pharmacy will succeed in this kind of social and economic milieu is precisely why I am here with you today. In this series of lectures, roundtables, and workshops, as so well thought out with your pharmacy leaders, we will get our arms around many of the vexing issues that we face together as clinical pharmacy administrators and practitioners. I hope that you will find the dialogue engaging, interesting, thought provoking, and a bit uncomfortable. When we feel some discomfort, we become aware that we are learning new methods and ways of seeing the world of pharmacy through the patient’s eyes and ears. It has been said many times, “People do not care how much you know, until they know how much you CARE.” In this spirit of inquiry and open discourse, we begin our journey together.

## 3. The Evolution of Patient-Driven Pharmacist Care

It is generally agreed that the clinical pharmacy movement in the United States began in the mid-1960s as a result of a number of drug-related systems and product innovations that required that pharmacists in organized healthcare settings become more involved in the management of individual patients’ drug therapies. A groundswell of interest worldwide became evident, as the impact of multi-national pharmaceutical manufacturing forged new alliances between the business and the science of pharmacy practice. Clinical pharmacy evolved over the ensuing decades as practice laws and regulations allowed pharmacists to directly engage in interactions with patients. Prior to that, it was considered unethical for a pharmacist to discuss or even label a prescription bottle with the name of a medication. If directions for use were not included in the prescription order, the pharmacist was not permitted to instruct the patient in the proper use of the medication. The typical “Take as directed” was translated from the Latin phrase, written in the prescription’s “Make thou” section, the words, “Ut Dictum” or “UD.” 

For clinical pharmacy, drug intelligence and patient “counseling” became hot topics in the 1970s. Led by Donald E. Francke [10], this new generation of pharmacists didn’t take “no” for an answer to their questions about drug therapy posed to physicians. They gathered literature to argue their points, and began to develop relationships with individual patients, albeit the major relationship was with a physician. At the bedside in major university medical centers like University of California–San Francisco and Long Beach Hospital [11], clinical pharmacists began to educate their teams with drug intelligence, and became indispensable members, the “go-to” professionals on drug therapy. The role that those early clinical pharmacists developed was blended; two parts clinical pharmacy, three parts drug distribution. These pharmacists ventured outside of the physical walls of the dispensary primarily because of the modeling of university pharmacy professors who were not content with filling and checking floor stock requisitions. Nor were they interested in being engaged remotely from the point of drug therapy decision-making. These pharmacists wanted to be where “the action is”, at the bedside and on rounds with others on the healthcare team. However, they understood very deeply that for pharmacy to succeed as a clinical discipline, the medications needed to be in the right place, at the right time, for the right reason, and in the right dose. While some didn’t, many held to the premise that pharmacy’s overall credibility to others—physicians, nurses, administrators, and each other—relied heavily on the integrity of the drug distribution system, especially regarding unit dose distribution and parenteral therapies like nutrition support and intravenous admixture services.

In the 1980s, intellectual and academic leaders in pharmacy began to question the profession’s future. Those such as Ken Barker, in his 1981 Harvey A.K. Whitney Lecture, reflected on what he called “pseudopharmacy practice,” and wondered whether we were really ready for a future where

TV users can push a button to request display of a dozen different data services, such as stock market quotations, ball scores, and theater reservations [where] home microcomputers will be as common as indoor plumbing.

[12]

While he didn’t define the construct, pseudopharmacy, as he would have done in one of his seminal medication error studies (i.e., using operational definitions), you get the gist of his concern. Pseudopharmacy means saying one thing, and doing another, often the opposite. It concerns integrity, honesty, and courage. “If we study it, and find that it’s good for patient care—that’s good pharmacy. If we just let it happen because nobody cares, that’s pseudopharmacy.” He asked what the future would be like for the profession based on evolving social trends, and made several prescient predictions. Among them: the outpatient pharmacy would become a joint venture between healthcare institutions and chain pharmacies; the inpatient pharmacy would become decentralized or what he called, “disassembled”; and “the pill machine will fill all routine prescription orders”. He posited that clinical pharmacists might even “service the patient’s implanted drug devices” at the bedside, but could “end up like the Maytag repairman, with little to do”, since the majority of drug distribution would be automated.

Then came the 1990s. Hepler and Strand, with Cipolle and Morley, coined the term, “pharmaceutical care” [13,14]. Pharmaceutical care is the patient-driven practice style of a new health professional. It is based on a societal need for a covenantal relationship between a patient and a practitioner. As they put it,

Pharmaceutical care is that component of pharmacy practice which entails direct interaction of the pharmacist with the patient for the purpose of caring for the patient’s drug-related needs.

[15]

Paraphrasing these sage colleagues and mentors, a professional practice is based on: (1) a vision of what one ought to do; (2) a mission specifying why one ought to do it; (3) a knowledge base from which one practices to achieve vision and mission; and (4) a management system that secures its consistent activation as a viable enterprise. To be considered a profession as such, there are several defining characteristics that differentiate it from other endeavors. In addition to the aforementioned, the hallmarks of a profession include: (1) autonomy of thought; (2) interdependent action; (3) self-regulation; (4) service-based; and (5) some form of goal-directedness. In the case of a professional practice devoted to pharmacotherapy, the nature, identification, and resolution of drug therapy needs and problems of individual patients.

## 4. What about Clinical Pharmacy, Disease State Management, and Consumerism Today?

Much of what I am saying next may scorch your sensibilities, and I am employing this risky tactic to get your attention and provoke your thought for what has gone unsaid about what has happened in pharmacy. It goes straight to vision and mission, as I believe that we must define ourselves, or others will. 

It can be said that between 1965 and 1985, the clinical pharmacy movement attempted to hoist pharmacy’s transition to professional practice by meeting drug therapy needs through the eyes and ears of the physician. It was a methodology for influencing the use of drugs in terms of what physicians perceived to be the drug-related problems. To some, clinical pharmacists became differentiated from other pharmacists in that they became the “super pharmacist,” the one who could do it all. Synthesize information, communicate with physicians, formulate drug policies and procedures, and manage drug budgets. The clinical pharmacist, compared to contemporaries, was the pharmacist that had some idea and working knowledge of the patient, but could only clarify and recommend rather than initiate and prescribe. The “mission” of clinical pharmacy in those days was, as McLeod held, “to maximize the contributions of pharmacists to patient care” [16]. In practice, clinical pharmacy was an eclectic mixture generated from doing the five rights of drug therapy use management. It had no independent basis of “right”, only the degree to which the physician accepted recommendations (right or wrong, no matter). Since other pharmacists wondered if the clinical one did anything (except to be at their beckon call to receive any potential problem), clinical activity was measured in terms of “interventions”. The operational definition of such was finding a prescriber’s error of omission or commission. These interventions were designed strictly to meet pharmacy needs, and not necessarily the patient’s needs. If a non-formulary drug was prescribed, if an unusual dosage form or mixture of drugs was ordered, if the medication orders entered by a ward secretary needed editing, if anything other than the placement of doses into a zip lock baggy was required, it was the job of the clinical pharmacist to solve. Real problems would go unresolved over the weekend if the clinical pharmacist failed to make contact with the pharmacy during off-hours. 

The clinical pharmacy movement effectively separated pharmacists into two groups: (1) those who thought about the problems of other professionals; and (2) those who did not think. A March 1983 issue of the Journal of the American Medical Association [17] hailed the clinical pharmacist with a picture on its cover, essentially as “the one in the pharmacy you can call about drug therapy because s/he knows what s/he is talking about”. (I am being severe in this regard to make a point.) The clinical pharmacy movement had a weak philosophical base, no consistent patient care process, and little, if any, management support.

If the clinical pharmacy movement could be characterized by an “uber pharmacist” meeting the drug related needs of physicians, the salient features of disease management pharmacy, in my mind, is that of a “carve-out pharmacist” meeting the needs of third party payers. Disease management has the appearance of a method of care, but, on closer inspection, it does not constitute a professional practice. It is a long distance, cost management technique for steering patients through the typical trajectory of a particular disease while minimizing expenses. In this rubric, the patient is guided through a series of clinical guidelines, critical pathways, and disease state algorithms to arrive at a pre-determined endpoint, the point at which they no longer need care nor cost the payer any money. To be fair, disease management has some intellectual appeal for minimizing expenses by reducing treatment variation. However, disease management often occurs without the patient’s knowledge, consent, or participation in care planning. Individual variation of need is minimized by design. 

At the macro level of analysis, in a 1996 address to the American Pharmaceutical Association’s 143rd annual meeting in Nashville, TN, USA, Professor T. Donald Rucker [18] identified a brief sampling of the problems of financing health care, centrally:

(1) Emphasis on integrating the health care delivery system more at the enrolment/market/management level than in the area of greatest need, clinical care.
(2) Continued fragmentation of medical records, and even elimination of encounter data, which impairs efforts to measure patient response and conduct meaningful quality assurance programs. Thus, we are still struggling to overcome Dr. Arnold Relman’s admonition in 1982 (NEJM)—‘it is the cost of ignorance that now become too steep for us to bear’. And
(3) Enhanced social expense and economic risk for patients through the proliferation of cost containment and cost shifting techniques, as well as growth in non-productive administrative expenses, or, as he said, in Washington lingo, the problem of smoke and mirrors.

He goes on to suggest the design features of a holistic health care system, outlining a “National Health Ensurance” or NHE, not NHI system. In them, he mentioned that “the concept of pharmaceutical care, as well as disease management programs and patient outcomes, might be expected to flourish with access to such a complete and efficient data base (and, conversely, to flounder in its absence”). In our present situation, over 20 years after his speech, health care is moving at warp speed towards the full manifestation of consumerism. The primary, yet unspoken, tenet of this movement is the separation of demand from supply in an environment driven by political pull and Congressional lobbying. Prices are not derived from free exchange. If we take the patient as a consumer of health services, several points become illuminated. Today, patients do not pay for health care; the government and/or their employers (if one has one) do. Patients have little choice of providers; often, they are locked into channels (sometimes by law) which require them to see doctors only, for these reasons only, for these tests only, for these drugs only, from these pharmacies only. The consumer neither pays for nor procures health care. Under these conditions, consumerism is flourishing because consumption does not “depend” on supply or price. 

## References

[1] Ernst F.R., Grizzle A.J. (2001). Drug-related morbidity and mortality: Updating the cost of illness model. J. Am. Pharm. Assoc..

[2] Kennedy A., Rogers A., Bower P. (2007). Support for self care for patients with chronic disease. Br. J. Med..

[3] Merks P., Kaźmierczak J., Olszewska A.E., Kołtowska-Häggström M. (2014). Comparison of factors influencing patient choice of community pharmacy in Poland and in the UK, and identification of components of pharmaceutical care. Patient Prefer. Adher..

[4] Kelly D.V., Young S., Phillips L., Clark D. (2014). Patient attitudes regarding the role of the pharmacist and interest in expanded pharmacist services. Can. Pharm. J..

[5] Stewart M., Brown J.B., Weston W.W., McWhinney I.R., McWilliam C.L., Freeman T.R. (2003). Patient-Centered Medicine: Transforming the Clinical Method.

[6] Fairhurst K., May C. (2001). Knowing patients and knowledge about patients: Evidence of models of reasoning in the consultation?. Fam. Pract..

[7] Binswanger H. (1992). Life-based teleology and the foundations of ethics. Monist.

[8] Lagnaoui R., Moore N., Fach J., Longy-Boursier M., Begaud B. (2000). Adverse drug reactions in a department of systemic diseases-oriented internal medicine: Prevalence, incidence, direct costs and avoidability. Eur. J. Clin. Pharmacol..

[9] Hallas J. (1996). Drug related hospital admissions in subspecialities of internal medicine. Dan. Med. Bull..

[10] Stevenson J.G., Beham R.E., Weber R.J. (2013). Profiles in leadership: Donald E. Francke, MSc, DSc (Hon). Hosp. Pharm..

[11] Smith W.E., Brodie D.C. (1980). The pharmacy service of Memorial Hospital Medical Center. Ann. Pharmacother..

[12] Barker K.N. (1982). Pseudopharmacy practice and the future. Am. J. Hosp. Pharm..

[13] Strand L.M., Morley P.C., Cipolle R.J., Ramsey R., Lamsam G.D. (1990). Drug-related problems: Their structure and function. DICP.

[14] Hepler C.D., Strand L.M. (1990). Opportunities and responsibilities in pharmaceutical care. Am. J. Hosp. Pharm..

[15] Cipolle R.J., Strand L.M., Morley P.C. (2012). Pharmaceutical Care Practice: The Patient-Centered Approach to Medication Management.

[16] McLeod D.C., Miller W.A. (1980). The Practice of Pharmacy: Institutional and Ambulatory Pharmaceutical Services.

[17] Lundberg G.D. (1983). The clinical pharmacist. JAMA.

[18] Unpublished manuscript supplied by T. Donald Rucker, Ph.D. For more information about Dr. Rucker. http://www.washingtonpost.com/wp-dyn/content/article/2007/01/30/AR2007013001727.html.

